# Suspension of the National Registry of Evidence-Based Programs and Practices: the importance of adhering to the evidence

**DOI:** 10.1186/s13011-018-0162-5

**Published:** 2018-06-22

**Authors:** Sharon Green-Hennessy

**Affiliations:** Department of Psychology, Loyola University Maryland, 4501 N. Charles Street, Baltimore, MD 21210 USA

**Keywords:** Evidence-based decision-making, Health policy, Health care services

## Abstract

Recently the United States Assistant Secretary of Mental Health and Substance Use disclosed having suspended the National Registry of Evidence-Based Programs and Practices, stating it was so deficient in both rigor and breadth that it must be replaced. However, a closer examination of her claims about the Registry indicates many of them to be inaccurate. Contrary to her assertions, the Registry is not devoid of medication-assisted treatments for opioid use; nor does it contain but a scant few interventions related to schizophrenia and psychosis. Moreover, many of her criticisms regarding rigor pertain to reviews completed since late 2015, when the Substance Abuse and Mental Health Services Administration altered key aspects of the Registry. In contrast to reviews generated under the 2007 rules, these newer reviews rely on fewer references, incorporate less expert input, are more likely to be based exclusively on gray literature, and are no longer required either to provide dissemination readiness information or meet certain minimum research quality standards. However, only 123 (25.7%) of the 479 Registry interventions have been reviewed solely using the problematic 2015 criteria, with the remaining 356 interventions having a review which use the 2007 guidelines. Yet, rather than address the agency’s recent missteps and expand the Registry’s content coverage, the agency appears to have decided to invest considerable resources into replacing it, relying heavily on expert consensus versus empirical data in its initial attempt to do so. This raises questions about the agency’s current commitment to evidence-based practice.

## Background

Recently, the Substance Abuse and Mental Health Services Administration (SAMHSA) announced the suspension and planned replacement of the National Registry of Evidence-Based Programs and Practices (NREPP) [1]. Any replacement would be SAMHSA's fourth attempt at providing evidence-based guidance, a move that could prove costly not just in dollars but credibility. Hence, before investing further in the creation of yet another system, it would appear prudent to examine the reported problems with NREPP.

### The 2007 version of NREPP

SAMHSA began NREPP in 1997 as a ranking of expert-recognized substance prevention programs, but quickly concluded a more rigorous process, inclusive of mental health and substance treatments, was needed [2]. After extensive scientific and public input, a radically different NREPP was unveiled in 2007. Contrary to its predecessor, NREPP of 2007 rejected the notion that a registry’s purpose was to tell its users what to do (a “forced fit” model); instead, it was designed as a decision support tool, integrating information into continuous quality and dissemination readiness ratings which could assist users in making informed decisions about which interventions best suited their specific needs (a “best fit” model) [2]. While not limiting itself to randomized controlled trials, the 2007 version required reviewed research meet certain minimum methodological standards [2]. Of note was its unique dual emphasis on efficacy and community effectiveness, with the latter embodied in its dissemination ratings, adverse effects notations, cultural adaptation descriptions, etc. [3].

### 2015 Revision

In 2015, SAMHSA changed NREPP back into a categorical ranking system [4]. NREPP’s distinctive array of numerical ratings was replaced with a “stoplight” outcome ranking (green-*Effective*, yellow-*Promising*, red-*Ineffective*), although exactly how these color-coded designations were derived was unclear [5]. To increase rigor, a Hedges *g* effect size statistic was introduced which allowed for findings across studies to be combined quantitatively, but at the same time, rigor was decreased by removing the requirement that studies meet certain specific minimum methodological requirements and limiting reviews to short-term outcome studies comparing interventions to an inactive or known less effective control [4, 6]. Finally, NREPP’s dissemination rating was removed, as was information on adverse effects, replications, and cultural adaptations [7].

At the time of its suspension, 479 interventions had been reviewed by NREPP. A total of 123 interventions (25.7%) had reviews using only the 2015 criteria, while 246 had only 2007 reviews (51.4%). As it had been SAMHSA’s intent to re-review all 2007 reviews, an additional 110 interventions (23.0%) had two reviews (both a 2007 and a 2015 one). Five hundred and seventy-seven of these reviews are currently posted on the NREPP website.

## Commentary

As Assistant Secretary McCance-Katz has claimed deficiencies in both rigor and breadth necessitate NREPP’s replacement [1], it is important to examine each of these stated concerns.

### NREPP’s rigor

McCance-Katz appears to have relied heavily on Gorman’s recent analysis of NREPP [8] when stating her concerns about the amount and quality of literature contained in each review. However, in her summary McCance-Katz fails to clarify that Gorman’s criticisms were of the 2015 reviews, not of the 2007 ones [8]. The distinction is an important one because the 2007 and 2015 reviews differ on more than just methodological features; they embody opposing philosophies of what a registry’s purpose should be. The 2007 reviews eschewed overall rankings because their intent was not to tell stakeholders what to do, but to inform them in making their own decisions as to which intervention would be most effective in their setting. To this end, they provided users with both internal and external validity data. Such an approach is consistent with research showing providers are more likely to implement evidence-based practices if they have a role in the adoption decision process [9, 10]. The 2015 reviews, in contrast, were designed to rank programs as being more effective for the population based primarily on internal validity and effect size; contextual and individual patient factors play little role in its criteria. Thus, the 2015 version of NREPP was, in effect, telling users these were the “best” interventions for them to use irrespective of their goodness of fit [11, 12]. Therefore, it is ironic that, despite their emphasis on internal validity, it is the 2015 reviews which have been criticized for their rigor [8].

Beginning in 2015 NREPP staff determined what materials NREPP reviewers would receive to evaluate [5], a change SAMHSA claimed would ensure a more comprehensive representation of the literature [4, 13]. However, an examination of the 110 interventions which have both a 2007 and 2015 review indicated that the 2015 quality assessments were based on significantly fewer references than the 2007 ones, *M* = 2.01 (*SD* = 1.40) vs. *M* = 3.02 (*SD* = 2.00), *t*(108) = − 5.57, *p* < .001, *d* = −.59. The 2015 reviews also contain fewer supplemental references (*M* = 2.29 (*SD* = 2.94) vs. *M* = 3.22 (*SD* = 2.87), *t*(103) = − 2.61, *p* = .01, *d* = −.32) and lacked the replications references which were a standard component of 2007 reviews (*M* = 1.75, *SD* = 2.82). Without considering the additional sources used in the 2007 dissemination ratings, the 2007 reviews, on average, were based on nearly twice as many references as the 2015 ones. Moreover, the 2015 reviews not only were based on less literature, but they were more likely to be based exclusively on “gray” literature than their 2007 counterparts (18.3% vs. 11.8%, Fisher’s exact test, *p* < .001) (S. Green-Hennessy, Ph.D., unpublished data, May 2018).

SAMHSA’s 2015 revisions compromised NREPP’s rigor in other ways as well. SAMHSA reduced the number of expert reviewers from four to one (re-reviews) or two (new reviews) [2, 4, 5], which is lower than the 2 to 13 typically used with national registries [14]. Moreover, as supporting research was no longer required to meet certain specific minimum methodological standards, the agency needed to create an additional *Inconclusive* outcome category to denote interventions whose research lacked sufficient rigor to generate an effect size [5]. SAMHSA framed the *Inconclusive* rating as a positive addition, stating its existence would dissuade the public from equating NREPP membership with NREPP endorsement [13]. However, as several programs which received an *Inconclusive* rating a year ago are currently describing themselves as being “listed on NREPP” (*Adult Self-Directed Learning Cognitive Lifeskills Program, Active Parenting of Teens: Families in Action, Reward and Reminder*) [15–17], the *Inconclusive* category does not appear to have addressed this concern. Lastly, the impact of the Hedges *g* statistic would have been greater if 48.1% of the outcomes in 2015 reviews were based on more than a single outcome measure. Of additional concern is that for 5.9% of the 2015 outcomes SAMHSA overtly cautions the user against using the Hedges *g* it supplies, noting significant study design weaknesses compromise its interpretability. Yet in 78.2% of those instances the intervention was still awarded a *Promising* rating.

Hence, McCance-Katz’ claim that NREPP reviews are regularly based on a single gray literature study [1] does not accurately characterize the 2007 reviews. While it is true that SAMHSA’s 2015 revision of NREPP was problematic, the majority of NREPP interventions have reviews using the 2007 criteria (*n* = 356; 74.3%).

### NREPP’s breadth

Assistant Secretary McCance-Katz also cited her inability to locate any medication-assisted therapies (MAT) for opioid use, and but a few interventions for schizophrenia, as being instrumental in her decision to suspend NREPP [1]. In reality, 26 (5.4%) of NREPP’s 479 interventions address opioid misuse and/or contain research demonstrating the intervention’s specific applicability to individuals with opioid use disorder (i.e., employment supports for those with this diagnosis); the majority of the 26 opioid interventions are medication assisted ones (61.5%). Moreover, 35 (7.3%) of NREPP’s interventions specifically target individuals classified as seriously mentally ill or who have been diagnosed with schizophrenia, schizoaffective, or bipolar disorder.

This is not to say that NREPP does not possess coverage gaps. As with other voluntary review systems [18], NREPP has grown unevenly. Irregular funding from SAMHSA forced the Registry to rely on developer self-nominations to help populate it early on [19], leading it initially to become disproportionately weighted towards proprietary prevention programs [20]. Increased funding to permit staff-initiated reviews and NREPP’s growing influence has increased the diversity of programs on NREPP [21]. Nevertheless, McCance-Katz sharply criticized NREPP for permitting developer self-nominations, claiming the practice precluded the Registry from being evidence-based [1], even though a recent review of national evidence-based registries indicated that 45% allow nominations [14].

Still, addressing gap areas, as well as creating a process for regular updating, had been identified by its developer as NREPP’s most important challenges going forward (K. D. Hennessy, Ph.D., personal oral communication, January 15, 2015). Nevertheless, it is important to note that NREPP operates within certain constraints such as those from the Food and Drug Administration (FDA); as noted on the NREPP website, the Registry does not evaluate freestanding pharmacological interventions [4, 22], as medication safety and efficacy traditionally fall within FDA’s purview. This limitation disproportionately affects certain substance and mental health disorders where pharmacological therapies are heavily utilized.

### SAMHSA's Evidence-Based Practice Resource Center

Instead of improving and expanding NREPP, SAMHSA has chosen to invest in an Evidence-Based Practices Resource Center [23], which it states exemplifies the agency’s new approach to evidence-based practice [24]. Of the 138 resources listed on the Center’s site in April 2018, nearly half (*n* = 66, 47.8%) were classified by SAMHSA as being expert consensus/guidelines, as opposed to empirical evidence, despite a recent Cochrane review which did not find expert mental health guidance to significantly influence which interventions practitioners employed [25].

Even more concerning is the looseness with which SAMHSA is now applying the “evidence-based” moniker. For instance, 25% (*n* = 11) of the 44 identified mental health resources listed on the Center’s website consist of recent SAMHSA generated webpages on various psychiatric disorders [23]. These pages identify certain treatments as being evidence-based, but do not contain a single reference justifying why those treatments, as opposed to others, are identified as such [26].

## Conclusion

In recent commentary piece, Gorman [8] asked if NREPP had lost its way. Unfortunately, the answer is yes. When NREPP lost its developer, it lost its way.

Moreover, SAMHSA has not sought stakeholder input to help it find its way again, with the Assistant Secretary failing to notify users that she had suspended the Registry until months after the fact [27]. Instead, the Assistant Secretary has chosen to discard a well-established, influential evidence-based system.

Equally puzzling is SAMHSA’s decision to replace NREPP with its Evidence-Based Practices Resources Center which is heavily populated by guidelines and contains a series of agency generated webpages which lack a single reference to justify their assertion that various mental health treatments are evidence-based. Such actions appear to be at odds with the 21st Century Cures Act, which mandates that substance and mental health prevention and treatment keep pace with science and that the Assistant Secretary provide on the agency’s website a listing of evidence-based practices whose evaluation metrics have been made publicly available [28].

While expert consensus guidelines may fill the gap when there is no applicable empirical evidence [29], there is evidence available which can help inform our work in the substance and mental health prevention and treatment fields. SAMHSA’s recent decisions though give rise to serious questions as to what criteria the agency is using currently to identify that evidence and if its own recent actions live up to the term “evidence-based.”

## Abbreviations

NREPP: National Registry of Evidence-Based Programs and Practices
SAMHSA: Substance Abuse and Mental Health Services Administration
FDA: Food and Drug Administration
